# Cutaneous metastatic adenocarcinoma complicated by spontaneous tumor lysis syndrome: A case report

**DOI:** 10.3892/ol.2014.2171

**Published:** 2014-05-23

**Authors:** YU WANG, CAIJUN YUAN, XIAOMEI LIU

**Affiliations:** 1 Department of Oncology, Graduate School of Liaoning Medical University, Jinzhou, Liaoning 121000, P.R. China; 2 Department of Oncology, The First Affiliated Hospital of Liaoning Medical University, Jinzhou, Liaoning 121000, P.R. China

**Keywords:** spontaneous tumor lysis syndrome, cutaneous metastatic adenocarcinoma

## Abstract

The present study reports the case of a 71-year-old female with metastatic adenocarcinoma of the skin who developed tumor lysis syndrome (TLS) upon admittance to the First Affiliated Hospital of Liaoning Medical University (Jinzhou, China). The patient presented to the hospital due to multiple subcutaneous nodules, lethargy and weakness, but succumbed without any cancer therapy. Metastases to the skin from solid carcinomas are uncommon, and several studies have reported patients with minimal primary symptoms despite extensive metastatic skin disease. However, few cases were accompanied with spontaneous TLS at the time of presentation. TLS may be a severe complication during the therapy for hematological and oncological diseases. Although spontaneous TLS in internal tumors has been reported, it is extremely rare. The present study highlights the fact that multiple subcutaneous metastases may occur with the symptoms of spontaneous TLS, and may be key for the early recognition of this syndrome.

## Introduction

Skin metastasis and tumor lysis syndrome (TLS) represent independent prognostic factors of poor survival in patients with malignant tumors. A typical cutaneous metastasis is in the form of a firm, painless papule or nodule and is a sign of underlying malignancy in 0.6–7.6% of cases (1–3). Additional clinical presentations include sclerodermoid, vascular, alopecic and erysipelas-like lesions (1). Delaying the progression of the disease, controlling the symptoms and maintaining a high quality of life for the patient are key to the successful treatment of the disease (4). TLS, characterized by severe hyperuricemia, hyperphosphatemia, hyperkalemia and hypocalcemia, is an oncological emergency due to massive tumoral cell lysis that usually presents following the initiation of chemotherapy, and only in extremely rare occasions develops in a spontaneous manner (5). However, the precise incidence of TLS is undefined and the standard therapy strategy for the treatment of TLS is based on volume expansion, decreasing metabolic abnormalities, and in the majority of cases, providing supportive treatment for renal failure (6,7).

There have been a few cases of spontaneous TLS described in the literature, however, there have been no reported cases of spontaneous TLS combined with multiple subcutaneous metastases. The present study reports a case of cutaneous metastatic adenocarcinoma with TLS that showed extremely rapid progression. The patient suffered from life-threatening complications, including TLS, liver failure and acute oliguria renal failure during the supportive treatment. Patient provided written informed consent.

## Case report

A 71-year-old female was admitted to the First Affiliated Hospital of Liaoning Medical University (Jinzhou, China) with multiple, red-colored, firm, non-tender subcutaneous nodules (0.5–6 cm in diameter) over the anterior chest wall, back, arms, inguinal region, neck, tongue and upper eyelid that had been present for 3 weeks. Upon examination the patient appeared lethargic and weak. The patient presented with a 2-year history of slight postmenopausal bleeding and an 11-month history of a mild sensation of suppression in the chest. The latter two symptoms were so mild that the patient had previously paid no attention to them.

**Figure 1:**
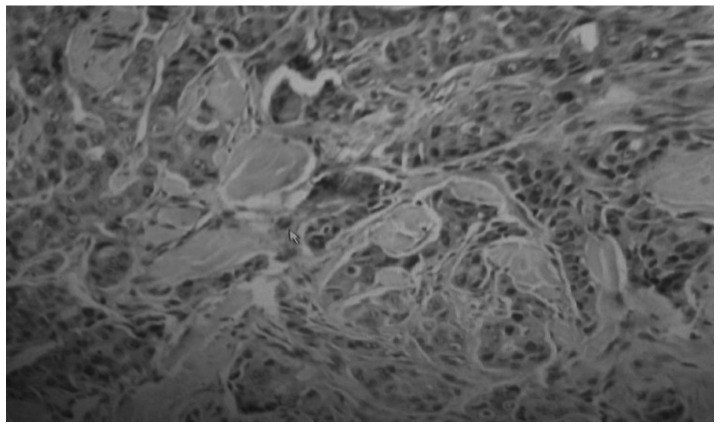
Biopsy of the nodules revealed metastatic adenocarcinoma, and the immunohistochemical profile was consistent with a digestive tract or ovary origin, showing the following expression results: Cytokeratin (CK)8(+), CK18(+), CK7(+), CK20(+), CK125(−), p63(−), gross cystic disease fluid protein-15(−), thyroid transcription factor-1(−), synaptophysin(−), chromogranin A(−) and hepatocyte paraffin 1(−).

**Figure 2:**
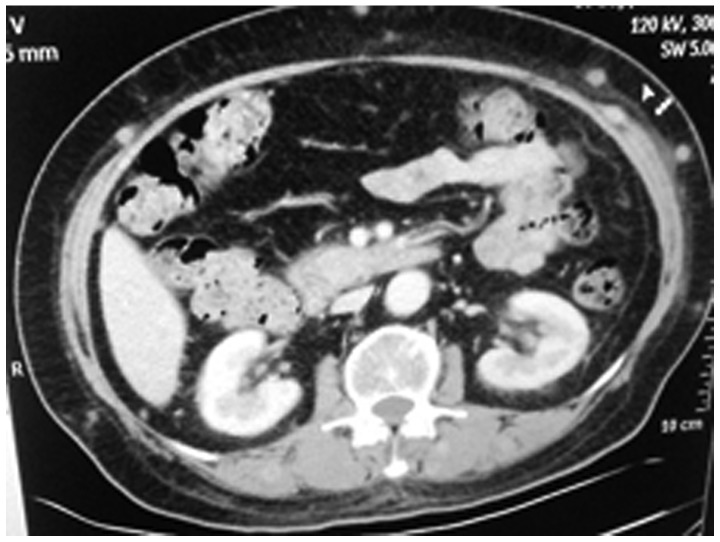
Enhanced abdominal CT on the day of admission revealing marked progression of the subcutaneous metastases.

Biopsies of these nodules revealed metastatic adenocarcinoma, and the immunohistochemical profile was consistent with a digestive tract or ovarian origin, showing positive expression results for cytokeratin (CK)8, CK18, CK7 and CK20, and negative results for CK125, p63, gross cystic disease fluid protein-15, thyroid transcription factor-1, synaptophysin, chromogranin A and hepatocyte paraffin 1 (Fig. 1). An enhanced abdominal computed tomography (CT) scan showed multiple subcutaneous metastases (Fig. 2) in the left kidney, right adrenal gland and liver. CT of the thorax revealed a solitary tubercle-like mass, 1 cm in diameter, at the inferior lobe of the right lung, and color doppler ultrasound of the pelvic cavity depicted no abnormalities.

The patient was referred to the First Affiliated Hospital of Liaoning Medical University for treatment of metastatic disease and due to serious weakness. Upon admission, the laboratory findings indicated leukocytosis (21.00×10^9^/l), anemia (113.00 g/l), thrombocytopenia (89.00×10^9^/l), hyperkalemia (5.78 mmol/l) and hyperphosphatemia (1.94 mmol/l), while the calcemia level was 2.44 mmol/l. The renal parameters were increased as follows: Creatinine, 112.87 μmol/l; urea, 21.22 mmol/l; uric acid, 616.00 μmol/l; and bicarbonate radical, 19.60 mmol/l. The liver parameters were: Alanine transaminase, 41.00 IU/l; aspartate transaminase, 65.00 IU/l; and alkaline phosphatase, 662.00 IU/l. The level of the majority of the tumor markers was markedly increased: Carcinoembryonic antigen, 254.30 ng/ml; cancer antigen (CA) 125, 3382.00 U/ml; CA72-4, 340.00 U/ml; CA19-9, 1480.00 U/ml; CA15-3, 94.30 U/ml; neuron-specific enolase, 66.05 ng/ml; and squamous cell carcinoma antigen, 345.70 ng/l. TLS was diagnosed and chemotherapy was delayed. The patient was supervised by cardiac monitor. Hemodialysis was not performed immediately as the patient’s family refused to consent to the treatment. Due to the change in electrolyte levels, a high level of uric acid and a low lever of bicarbonate radical, high doses of allopurinol, calcium and potassium-binders were administered intravenously. Bicarbonate was administered to compensate for metabolic acidosis. However, the patient became progressively more tachypneic. In the second week of hospitalization, the patient developed tachycardia, with a pulse rate of 130 beats/min, and hypotension, with a systolic blood pressure of 70 mmHg and a diastolic blood pressure of 50 mmHg. Despite cardiopulmonary support, the patient died of acute anuria renal failure at the end of the second week, with general edema occurring at the end.

## Discussion

To the best of our knowledge, cutaneous metastases as distant metastases, often appear subsequent to the original symptoms. The present case proved to be an exception in several ways. The patient had minimal original symptoms despite suffering from metastatic skin disease, and the primary origin could not conclusively be determined prior to mortality. The mechanism of distant metastasis, including cutaneous metastasis, is not fully understood. Cutaneous metastasis occurs mainly via the hematogenous and lymphatic routes, and it is indicative of an extremely advanced stage, with a poor prognosis. With regard to the patient, attempts at treatment remain unsatisfactory and difficult (8,9).

Acute TLS is an life-threatening condition characterized by severe hyperuricemia, hyperphosphatemia, hyperkalemia, hypocalcemia, increased anion gap metabolic acidosis and acute renal failure (10). TLS has been described as a rare event, complicating the treatment of aggressive hematological tumors (11). In solid tumors, TLS is even more rare, and it has been reported to occur subsequent to therapy. Only a few cases of spontaneous TLS in solid tumors have been described (12–15). In the present patient, TLS appeared at the time of admission, without any therapy having previously been provided.

In the patient of the present study, the prognosis was made worse by multiple general subcutaneous metastatic nodules, with multiple organ metastases and high uric acid, serum potassium ion and tumor marker levels, without serious original symptoms prior to the evident weakness. Multiple metastases to the skin may also be key for the early recognition of TLS, along with the elevation of uric acid, serum potassium ion and phosphorus levels, and acute oliguria renal failure. Renal impairment may have been intensified by the nephrotoxic contrast material used during the supportive treatment.

In conclusion, spontaneous TLS may develop during the course of multiple cutaneous metastases as an atypical presentation, and despite intensive treatment, the syndrome can lead to fatality, particularly in elderly individuals. In a previous study, all patients >60 years of age with acute spontaneous TLS succumbed shortly after presentation (16). Oncologists should be aware of the potential complications presented in the present study of multiple subcutaneous metastases accompanied with TLS, for the treatment of advanced tumors with rapidly progressive and high-volume choriocarcinoma. Further studies are required to elucidate the mechanisms behind cutaneous metastasis and spontaneous TLS at the molecular level, and to analyze potential molecular biomarkers in order to identify which patients are most likely to develop spontaneous TLS. We believe that it is important to make such rare cases known and also to identify a breakthrough therapy for advanced malignant tumors.
